# Surface Exposure and Packing of Lipoproteins into Outer Membrane Vesicles Are Coupled Processes in *Bacteroides*

**DOI:** 10.1128/mSphere.00559-18

**Published:** 2018-11-07

**Authors:** Ezequiel Valguarnera, Nichollas E. Scott, Philippe Azimzadeh, Mario F. Feldman

**Affiliations:** a Department of Molecular Microbiology, Washington University in Saint Louis, Saint Louis, Missouri, USA; b Department of Microbiology and Immunology, The University of Melbourne, Victoria, Australia; University of Iowa

**Keywords:** *Bacteroides*, OMV, hydrolases, lipoproteins, vesicles

## Abstract

Outer membrane vesicles (OMVs) are spherical structures derived from the outer membranes (OMs) of Gram-negative bacteria. *Bacteroides* spp. are prominent components of the human gut microbiota, and OMVs produced by these species are proposed to play key roles in gut homeostasis. OMV biogenesis in *Bacteroides* is a poorly understood process. Here, we revisited the protein composition of Bacteroides thetaiotaomicron OMVs by mass spectrometry. We confirmed that OMVs produced by this organism contain large quantities of glycosidases and proteases, with most of them being lipoproteins. We found that most of these OMV-enriched lipoproteins are encoded by polysaccharide utilization loci (PULs), such as the *sus* operon. We examined the subcellular locations of the components of the Sus system and found a split localization; the alpha-amylase SusG is highly enriched in OMVs, while the oligosaccharide importer SusC remains mostly in the OM. We found that all OMV-enriched lipoproteins possess a lipoprotein export sequence (LES), and we show that this signal mediates translocation of SusG from the periplasmic face of the OM toward the extracellular milieu. Mutations in the LES motif caused defects in surface exposure and recruitment of SusG into OMVs. These experiments link, for the first time, surface exposure to recruitment of proteins into OMVs. We also show that surface-exposed SusG in OMVs is active and rescues the growth of bacterial cells incapable of growing on starch as the only carbon source. Our results support the role of OMVs as “public goods” that can be utilized by other organisms with different metabolic capabilities.

**IMPORTANCE** Species from the *Bacteroides* genus are predominant members of the human gut microbiota. OMVs in *Bacteroides* have been shown to be important for the homeostasis of complex host-commensal relationships, mainly involving immune tolerance and protection from disease. OMVs carry many enzymatic activities involved in the cleavage of complex polysaccharides and have been proposed as public goods that can provide growth to other bacterial species by release of polysaccharide breakdown products into the gut lumen. This work shows that the presence of a negatively charged rich amino acid motif (LES) is required for efficient packing of the surface-exposed alpha-amylase SusG into OMVs. Our findings strongly suggest that surface exposure is coupled to packing of *Bacteroides* lipoproteins into OMVs. This is the first step in the generation of tailor-made probiotic interventions that can exploit LES-related sequences to generate *Bacteroides* strains displaying proteins of interest in OMVs.

## Abstract (precis)

Species from the *Bacteroides* genus are predominant members of the human gut microbiota. OMVs in *Bacteroides* have been shown to be important for the homeostasis of complex host-commensal relationships, mainly involving immune tolerance and protection from disease. OMVs carry many enzymatic activities involved in the cleavage of complex polysaccharides and have been proposed as public goods that can provide growth to other bacterial species by release of polysaccharide breakdown products into the gut lumen. This work shows that the presence of a negatively charged rich amino acid motif (LES) is required for efficient packing of the surface-exposed alpha-amylase SusG into OMVs. Our findings strongly suggest that surface exposure is coupled to packing of *Bacteroides* lipoproteins into OMVs. This is the first step in the generation of tailor-made probiotic interventions that can exploit LES-related sequences to generate *Bacteroides* strains displaying proteins of interest in OMVs.

## INTRODUCTION

Outer membrane vesicles (OMVs) are small spherical structures derived from the outer membranes (OMs) of most Gram-negative bacterial species. OMVs are composed of phospholipids, lipopolysaccharide (LPS), or lipooligosaccharide (LOS) and OM and periplasmic proteins (1, 2). OMVs can mediate host-microbe interactions by facilitating long-distance delivery of virulence factors, by modulating the host immune response, and by contributing to antibiotic resistance (1, 3–8). Despite these key roles in bacterial physiology, OMV biology is poorly understood. Recent research indicates that OMVs are produced by diverse mechanisms. For example, OMVs are proposed to be generated by LPS remodeling in Porphyromonas gingivalis, Salmonella enterica, and Pseudomonas aeruginosa (9–12). In contrast, OMVs from Haemophilus influenzae and Vibrio cholerae are thought to be the result of an accumulation of phospholipids in the OM outer leaflet mediated by their specialized VacJ/Yrb transporter (13). Thus, it appears that there is not a universal mechanism of OMV biogenesis. For most species, including *Bacteroides* spp., OMV biogenesis remains poorly understood (5).

Species from the phylum *Bacteroidetes* compose a major part of the human gut microbiota (14, 15). OMVs from these organisms are proposed to play important roles in the commensal-host relationship, including the delivery of immunomodulatory molecules to host immune cells, an interaction that appears to help prevent colitis flare-ups in the context of inflammatory bowel disease (IBD) (7, 8). Furthermore, *Bacteroides* OMVs have been proposed to interfere with intracellular Ca^2+^ signaling in host cells (16). Most studies focus primarily on two predominant species in the human gut, Bacteroides thetaiotaomicron and Bacteroides fragilis. These species produce large amounts of uniformly sized OMVs that have a protein composition distinct from that of the OM, indicating that these OMV particles are not by-products of bacterial lysis (5). Most *Bacteroides* OMV-exclusive proteins are putative acidic lipoproteins with hydrolase activity, suggesting that proteins with similar structural and physicochemical properties are selectively sorted to OMVs (5). Many *Bacteroides* enzymatic lipoproteins are encoded on polysaccharide utilization loci (PULs), which constitute ∼20% of the B. thetaiotaomicron genome and are essential for the breakdown and acquisition of plant, fungus, and mucin complex polysaccharides (17–19). PULs typically consist of at least one TonB-dependent receptor, or SusC-like protein, and one nutrient binding accessory protein, or SusD, and they can also present other accessory proteins (20). PULs can also carry two-component systems that sense nutrient variations in the medium, with subsequent induction of polysaccharide utilization genes required for the utilization of complex carbon sources (17–19, 21–24). Hence, *Bacteroides* cells can modify the enzymatic compositions of OMVs according to available carbon sources (5). The enzymatic arsenal carried by *Bacteroides* OMVs appears to carry a “social” function, as the products of OMV-mediated hydrolysis can be utilized by other bacteria within the gut (25, 26).

Here, we further characterized the protein composition of B. thetaiotaomicron OMVs. We confirmed that OMVs produced by this organism contain mainly putative acidic lipoproteins with hydrolytic or carbohydrate-binding activities. Many of these OMV-enriched lipoproteins were found to be encoded by PULs. We examined the subcellular localization of the components of the archetypical PUL, the starch utilization system (Sus) (20, 27), and found that the alpha-amylase SusG and other Sus lipoproteins are highly enriched in OMVs. In contrast, the oligosaccharide importer SusC remains mostly in the OM, as previously characterized (20, 27, 28). We show that the presence of a lipoprotein export sequence (LES) mediates translocation of SusG from the periplasmic face onto the extracellular milieu and is both required and sufficient for SusG to localize preferentially to OMVs. Our results support the role of OMVs as “public goods” that can be utilized by other organisms with different metabolic capabilities.

## RESULTS

### Proteomics analysis of membrane and OMV fractions from B. thetaiotaomicron.

**FIG 1:**
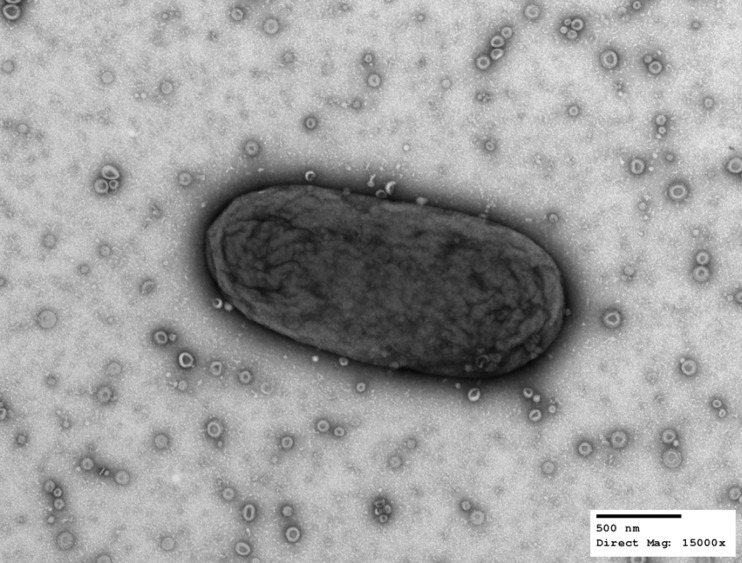
B. thetaiotaomicron produces outer membrane vesicles (OMVs). Transmission electron microscopy of a single B. thetaiotaomicron cell together with OMVs in the extracellular milieu. B. thetaiotaomicron cells were swabbed from a solid-medium plate, suspended in PBS, and processed for TEM. Images were acquired at a direct magnification of ×15,000. The scale bar represents 500 nm. (Image courtesy of Wandy Beatty, Molecular Microbiology Imaging Facility, WUSTL.)

Electron micrograph analysis confirmed that B. thetaiotaomicron produces large amounts of uniform OMV particles (Fig. 1). We previously performed a proteomic analysis of B. thetaiotaomicron OMs and OMVs by employing Triton X-100 for the purification of OM proteins (5). In this work, we employed *N*-lauroylsarcosine (Sarkosyl), which has been widely used for the separation of inner membrane (IM) and OM fractions in Gram-negative bacteria (29–31) to verify that the apparent selective fractionation of proteins into OM and OMV was not due to the use of a specific detergent. Using early-stationary-phase cultures from B. thetaiotaomicron, we prepared the different membrane fractions as indicated in Fig. S1 in the supplemental material. In our previous study, OMVs were not treated with the detergent employed to extract the IM proteins from total membrane preparations. To rule out possible detergent effects, we added an additional step consisting of incubating OMVs in 1% Sarkosyl prior to the ultracentrifugation of the samples to recover an OMV supernatant (OMV-S) and an OMV pellet (OMV-P). Samples were lyophilized for proteomic analysis by liquid chromatography-tandem mass spectrometry (LC-MS/MS), and aliquots were visualized by Coomassie blue staining (Fig. S1).

10.1128/mSphere.00559-18.1 FIG S1 Protocol for purification and analysis of B. thetaiotaomicron OMV and membrane fractions. (a) We performed membrane and OMV fractionation as indicated using cultures grown overnight in TYG medium. Fractions were incubated with 1% Sarkosyl for 1 h at RT and ultracentrifuged for 2 h at RT. Fractions were resuspended in 50 mM HEPES, pH 7.4. (b) Aliquots from each fraction were analyzed by SDS-PAGE and Coomassie blue staining. Fractions labeled as OM (green) and OMV-P (blue) are the most relevant fractions for comparison by MS. Download FIG S1, TIF file, 2.2 MB. Copyright © 2018 Valguarnera et al. 2018 Valguarnera et al. This content is distributed under the terms of the Creative Commons Attribution 4.0 International license.

**FIG 2:**
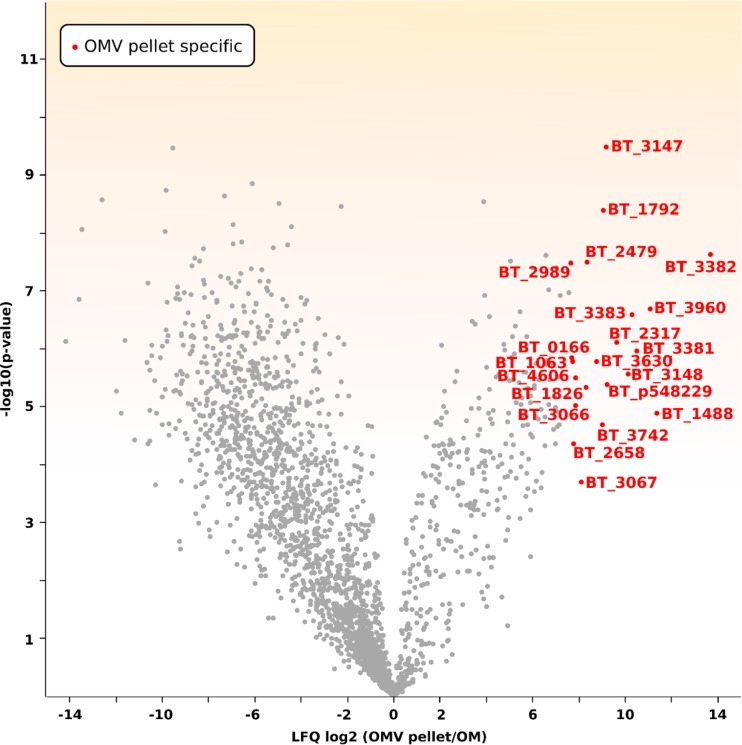
The B. thetaiotaomicron OMV pellet (OMVp) protein content is different from that of the OM. Three hundred micrograms of each preparation and biological replicates of purified OM and OMVp proteins were digested with trypsin. The resulting peptides were enriched and then analyzed via liquid chromatography coupled to tandem mass spectrometry (LC-MS/MS) as explained in Materials and Methods, followed by protein identification with the Mascot search engine using the UniProt database. Volcano plot shows OM and OMVp protein populations. Red labels indicate the proteins with the highest OMVp enrichment in comparison to that of OM.

**FIG 3:**
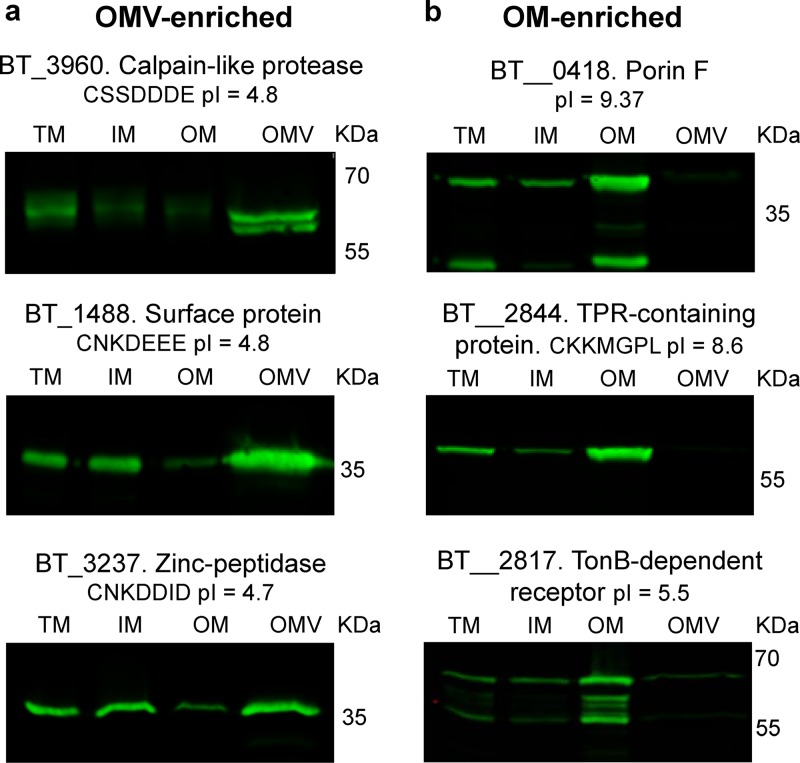
Validation of OMV- and OM-enriched proteins. Candidate ORFs that encode proteins identified in our MS analysis as OMV or OM enriched were cloned into pFD340 with a C-terminal 6×His tag. Constructs were introduced into B. thetaiotaomicron by conjugation, generated strains were grown in TYG medium, and fractions were prepared. Ten micrograms of each fraction was subjected to 12% SDS-PAGE and analyzed by Western blotting using anti-His polyclonal antibodies. (a) OMV-enriched proteins; (b) OM-enriched proteins. The isoelectric point as well as the residues following the lipoprotein attachment cysteine (for lipoproteins) are indicated below the protein name.

Our new data set with annotations and predicted functions and locations for proteins in all fractions is provided as Table S1 in the supplemental material. We confirmed our previous findings showing an enrichment of lipoproteins in the OMV fraction in comparison with their level in the OM fraction (Table 1; Table S2) (5). Figure 2 highlights the top enriched proteins in the OMV. We found that 18 out of the 23 OMV-exclusive lipoproteins from our previous study are also enriched in the new OMV preparations (5). We then cloned three OMV- and three OM-enriched proteins identified in our studies into the expression vector pFD340 as C-terminal 6×His-tagged proteins to confirm their localization by Western blotting. Figure 3a shows that a putative cell surface protein (BT_1488), as well as a putative calpain-like protease (BT_3960) and a putative zinc peptidase (BT_3237), is highly enriched in OMVs. Figure 3b shows that BT_0418, BT_2844, and BT_2817, identified as OM-enriched proteins by MS, are retained at the OM and are not present in OMVs. BT_0418 is a porin F ortholog (8-β-strand protein with a peptidoglycan-binding domain), BT_2817 is a TonB-dependent receptor (22-β-strand protein), and BT_2844 is a lipoprotein-containing tetratricopeptide repeat (TPR) motif. Both BT_0418 and BT_2817 are not predicted to be lipoproteins, they carry a signal peptidase I (SPI) cleavage site, and they do not contain the lipoprotein attachment cysteine residue. Before cloning this group of OMV- and OM-enriched proteins, we observed that the automated start codon annotation for some open reading frames (ORFs) (BT_0418 and BT_2844) seemed incorrect. Any mistakes in start codon-predicted annotations were corrected accordingly (see Materials and Methods). These sets of experiments demonstrate that lipoproteins are indeed differentially sorted between OMs and OMVs.

**TABLE 1:**
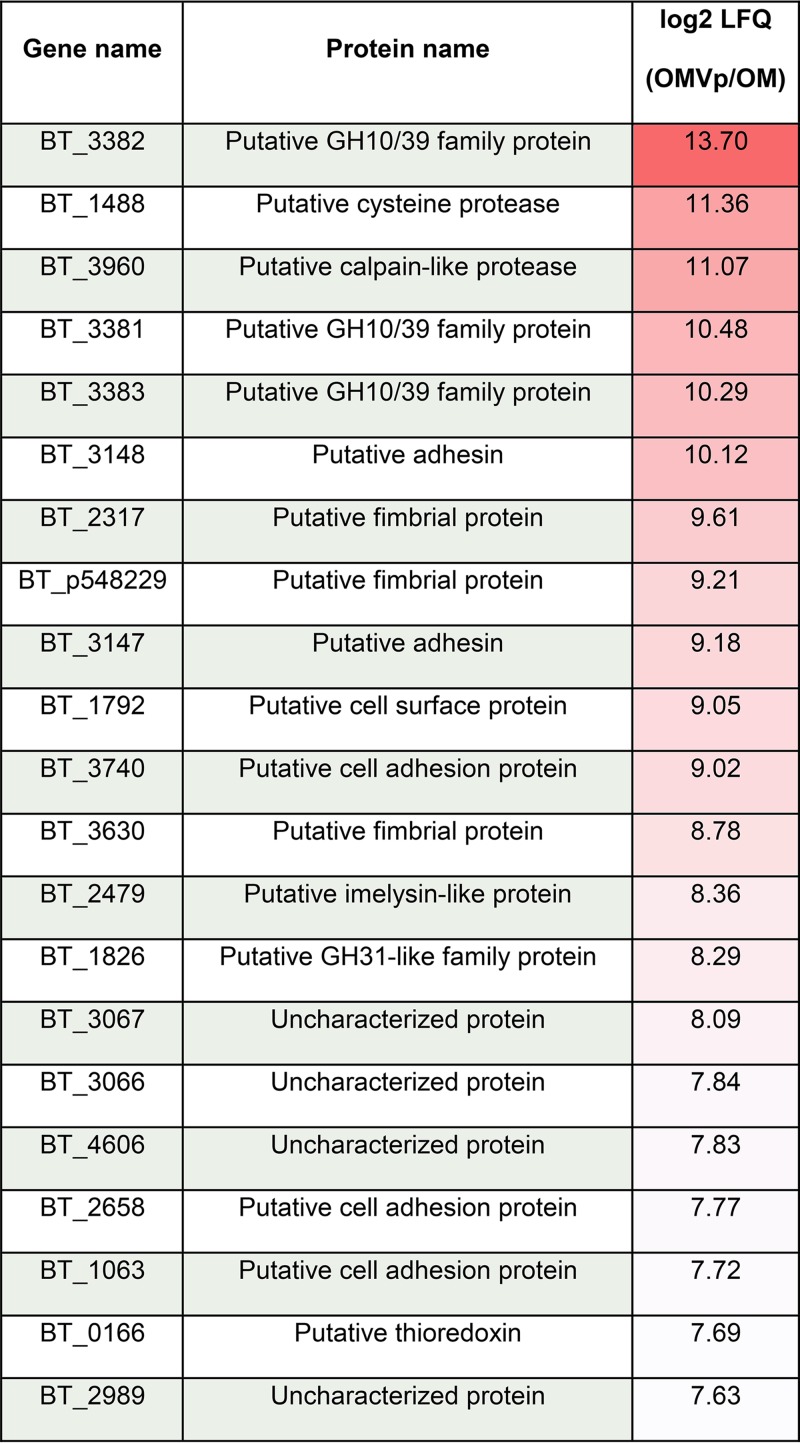
Top 20 most OMVp-enriched proteins, including protein name and enrichment value^*a*^

a LFQ, label-free quantification.

10.1128/mSphere.00559-18.5 TABLE S1 Complete MS data set. Download Table S1, XLSX file, 2.1 MB. Copyright © 2018 Valguarnera et al. 2018 Valguarnera et al. This content is distributed under the terms of the Creative Commons Attribution 4.0 International license.

10.1128/mSphere.00559-18.6 TABLE S2 OMV- and OM-enriched proteins. Download Table S2, XLSX file, 0.1 MB. Copyright © 2018 Valguarnera et al. 2018 Valguarnera et al. This content is distributed under the terms of the Creative Commons Attribution 4.0 International license.

### Common features of OMV-enriched lipoproteins.

**FIG 4:**
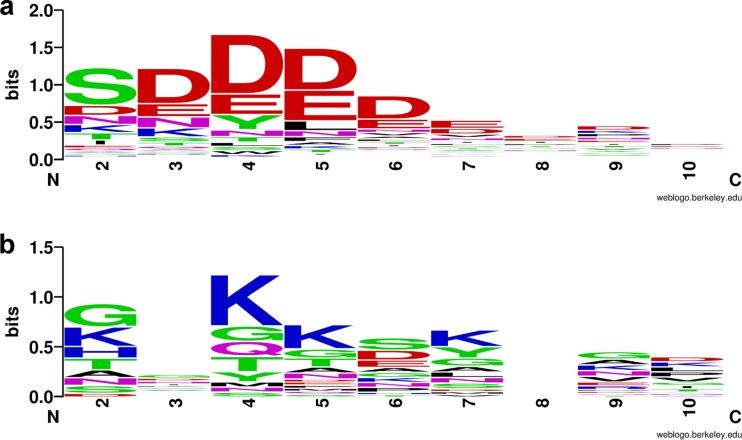
OMV-enriched proteins show a conserved N-terminal LES motif. The top 91 OMV-enriched proteins (a) and top 24 OM-enriched proteins (b) were aligned using the lipoprotein attachment cysteine at the +1 position (not shown in the logo), followed by the 9 C-terminal contiguous residues. The lipoprotein export sequence (LES) consensus was generated using WebLogo (https://weblogo.berkeley.edu/logo.cgi) (56).

Consistently with our previous results, we found that lipoproteins enriched in OMVs are acidic, with an average isoelectric point of 4.86 (5). We also determined that OMV-enriched lipoproteins possess a negatively charged rich amino acid motif, S(D/E)_3_, adjacent to the cysteine residue required for lipoprotein attachment (Fig. 4; Table S2). A similar motif [K-(D/E)_2_ or Q-A-(D/E)_2_] in the oral pathogen Capnocytophaga canimorsus, a member of the *Bacteroidetes* phylum, has recently been described. The report showed that this motif functions as a lipoprotein export signal (LES) required for surface exposure of OM lipoproteins (32). The authors also showed that proteins carrying a LES from B. fragilis were exposed when expressed in *C. canimorsus*. We found that many OMV-enriched B. thetaiotaomicron lipoproteins are putative protein and sugar hydrolases required for the breakdown of complex nutritional sources. The presence of a LES on OMV-enriched proteins is consistent with their annotated functions, as they are required to face the extracellular milieu to access their cognate substrates. Conversely, OM-enriched lipoproteins like BT_2844 do not carry a LES motif. These proteins have lower sequence conservation and lower frequency of negatively charged amino acids along the residues adjacent to the lipoprotein attachment cysteine and are therefore expected to be oriented toward the periplasmic face of the OM (Fig. 4b; Table S2).

### SusG and other Sus lipoproteins are enriched in OMV, and SusC is retained at the OM.

**FIG 5:**
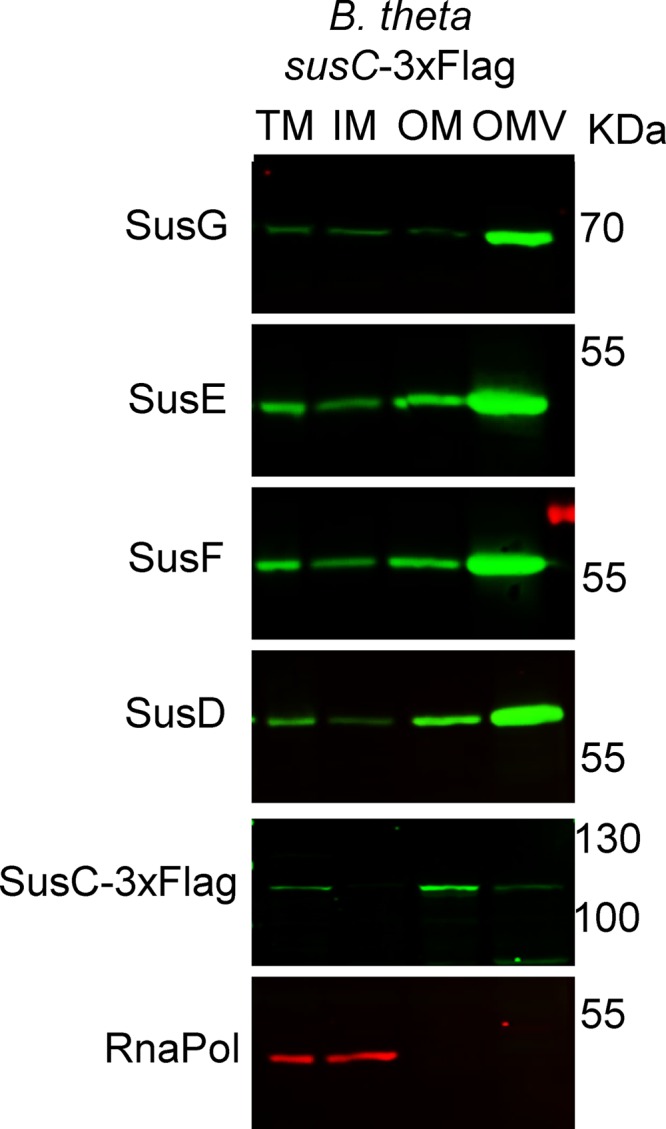
Sus lipoproteins are OMV enriched, while SusC is OM enriched. B. thetaiotaomicron cells containing a tagged genomic copy of *susC* with a 3×Flag tag were grown in TYM medium (TYG recipe with 0.5% maltose instead of glucose for induction of the *sus* operon). Fractions were prepared and analyzed by 12% SDS-PAGE and Western blotting using specific anti-Sus, anti-Flag, and anti-RNApol antibodies.

In B. thetaiotaomicron, hydrolytic lipoproteins are encoded mainly in PULs. PUL and PUL-like operons account for approximately 20% of the B. thetaiotaomicron genome. Thirty-six lipoproteins encoded in PUL and PUL-like operons were found in our OMV-enriched proteins. One of the most studied lipoproteins from B. thetaiotaomicron is the α-amylase SusG, encoded by the *sus* operon and essential for starch catabolism (33, 34). The *sus* operon has been shown to be induced by starch and maltooligosaccharides (35). Our MS data show that even under noninducing conditions, two lipoproteins encoded by the *sus* operon, SusD and SusE, are enriched in OMVs (Table S2). Given the importance of starch in the mammalian diet, we investigated the subcellular localization of the components of the Sus operon. We found that all Sus lipoproteins, in particular SusG, are enriched in the OMV. The only exception was the porin SusC, which is retained mostly at the OM (Fig. 5). We also observed considerable levels of SusD in the OM, concomitant with its strong functional interaction with SusC (28). This result suggests that PUL components can exert their biological effect not only at the level of the outer membrane but also through OMVs. These findings represent a localization split for the components of the *sus* operon exclusively and need to be further validated for other *sus*-like systems through the B. thetaiotaomicron genome, especially because we observed other SusC-like proteins enriched in both OMs and OMVs (Table S2).

### LES is required for SusG exposure and packing into OMVs.

**FIG 6:**
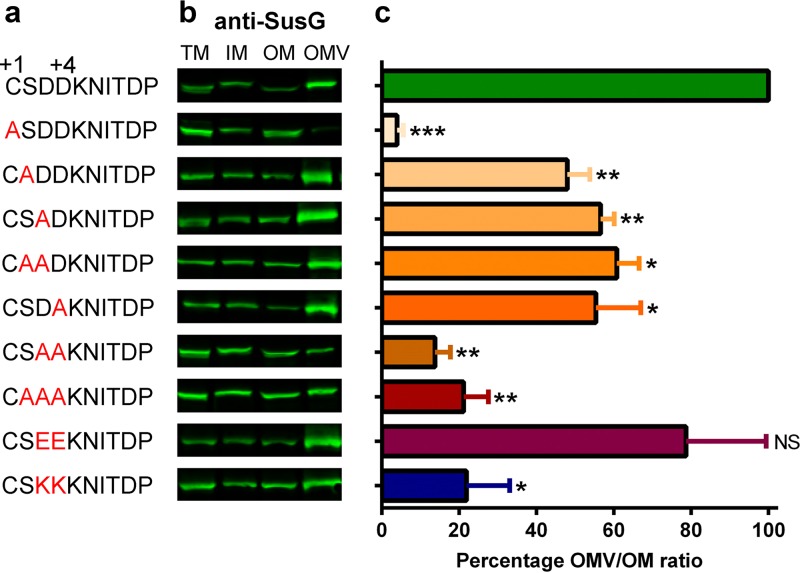
SusG LES is required for efficient packing into OMVs. (a) SusG LES point mutants were generated on pFD340/*susG*-6×His using Quick site-directed mutagenesis. (b) Constructs were introduced in the Δ*susG* background, and generated strains were grown in minimal medium with glucose as the only carbon source. Fractions were prepared and analyzed by SDS-PAGE and Western blotting using anti-SusG antibodies. (c) OMV/OM ratios were calculated using the fluorescence signal values for each protein and are plotted as a percentage of the WT OMV/OM ratio (100%). Statistical significance was determined by unpaired *t* test of each LES variant in comparison with values for the wild-type LES strain. *, *P* values < 0.03. NS, not significant. The experimental results are representative of those of three biological replicates; shown are mean values with standard deviations (SD) for two technical replicates.

All B. thetaiotaomicron endoglycanases involved in the first step of polysaccharide breakdown, including SusG, are surface-exposed lipoproteins (33, 36). Because all OMV-enriched lipoproteins contain a LES motif, we investigated the link between surface exposure and packing into OMVs. SusG contains only two Asp residues following the +2 Ser residue, and therefore, for the purpose of this work, we provisionally define its LES motif as CSDD. We performed a mutational analysis on the LES motif of SusG and analyzed the localization of the protein by fractionation and Western blotting (Fig. 6). We cloned wild-type (WT) His-tagged SusG and a set of SusG derivatives carrying amino acid replacements in the LES (Fig. 6a) and expressed them in the Δ*susG* strain (37). Replacement of the lipid attachment site (C23) by Ala resulted in abrogation of SusG recruitment into OMVs. Furthermore, substitution of a single Asp residue with Ala (CSAD, CSDA, CAAD) decreased OMV packaging (∼50% relative to that in the WT), while mutation of both Asp residues by either Ala or Lys (CSAA, CAAA, CAKK) had a more dramatic effect (∼15% relative to that of the WT) (Fig. 6c). Conservative replacement of Asp by Glu (CSEE) displayed a WT-like behavior. These results indicate that the LES motif CS(D/E)_2_ is required for SusG packaging into OMVs.

**FIG 7:**
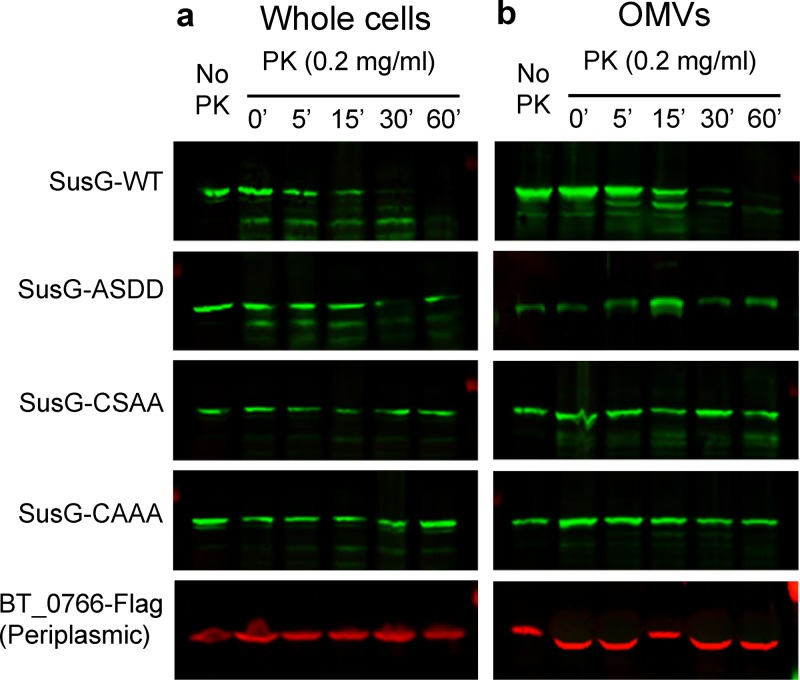
The LES is required for SusG surface exposure. WT SusG and the LES mutants were assessed for their surface exposure by proteinase K assays using whole cells (a) and OMVs (b). Proteinase K (PK) was added and incubated at 37°C; at different times (in minutes), aliquots were TCA precipitated and analyzed by SDS-PAGE and Western blotting using anti-SusG antibodies. We used 3×Flag-tagged BT_0766, a periplasmic soluble protein found in OMVs, as an outer membrane and OMV integrity control.

The LES motif has been defined as a surface exposure tag in *C. canimorsus* (32). We used the WT LES SusG construct as well as the non-Asp variants (CSAA, CAAA) to determine whether this motif is also required for surface exposure in B. thetaiotaomicron. Whole cells and OMVs from the different strains were subjected to proteinase K (ProK) sensitivity analysis. Only the WT SusG was degraded by ProK (Fig. 7). As a control, we employed a periplasmic soluble protein that localizes into the lumens of OMVs (BT_0766) and is therefore protected from ProK degradation. Taken together, these experiments confirm that LES mediates both SusG surface exposure and enrichment in OMVs.

### OMVs containing SusG rescue the Δ*susG* strain’s growth in starch.

**FIG 8:**
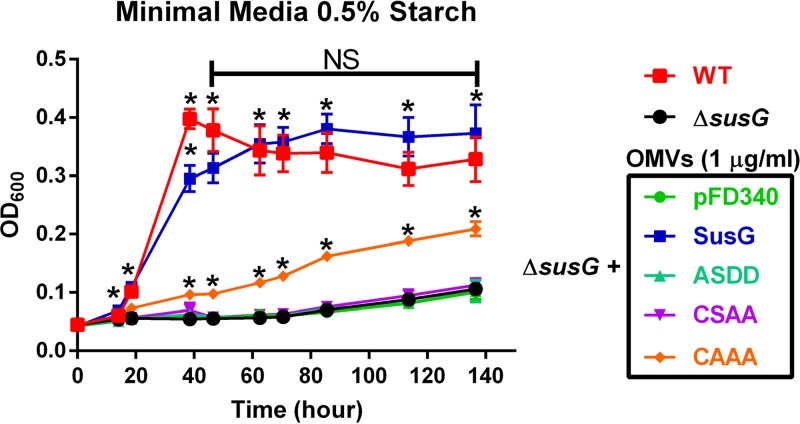
OMVs displaying WT SusG can rescue a strain with a Δ*susG* growth phenotype on starch as the carbon source. WT and Δ*susG* strains were grown overnight in TYG medium. Cultures were washed with minimal medium without any carbon source and normalized by OD. Minimal medium with 0.5% starch as the only carbon source was inoculated with WT or Δ*susG* strains to a final OD_600_ of 0.05. OMVs purified from the Δ*susG* strain containing different pFD340 derivatives were added to the Δ*susG* cultures in minimal medium with starch (1-µg/ml final concentration of OMVs). Aliquots were taken at different times, and the OD_600_ was measured to determine growth. Statistical significance when two growth curves were compared was determined by performing one pair of an unpaired *t* test analysis per each time point. *, *P* values < 0.01. The experiment is representative of three biological replicates; shown are mean values with SD for three technical replicates.

Our experiments demonstrated that SusG is surface exposed and packed into OMVs. OMVs carrying certain glycosyl hydrolases can digest complex polysaccharides, providing essential nutrients to bacteria unable to degrade these substrates (25, 26). The Δ*susG* strain is unable to grow on minimal media with starch as the sole carbon source (Fig. 8). We investigated whether surface exposure of OMV-delivered SusG can rescue the Δ*susG* growth phenotype on starch. For this, we employed OMVs produced by the Δ*susG* strain expressing WT SusG and SusG with a mutated LES (ASDD, CSAA, and CAAA). The ASDD mutation renders soluble SusG in the periplasm, while the CSAA and CAAA SusG variants face the periplasmic side of the OM (Fig. 7). Purified OMVs were added to the Δ*susG* strain in minimal media containing starch. Only WT OMVs restored the growth of the Δ*susG* strain to wild-type levels (Fig. 8). OMVs from the *susG* deletion-carrying vector control (pFD340) or expressing the nonlipoprotein mutant (ASDD) or the CSAA mutant were unable to rescue the expression of SusG, while the CAAA mutant displayed an intermediate phenotype. All together, our results indicate that OMVs carrying only surface-exposed SusG can mediate cross-feeding of other bacteria.

## DISCUSSION

The human gut microbiome is composed largely of species from the *Bacteroides* genus. *Bacteroides* spp. are important for gut homeostasis (14, 15). They establish ecological interactions with each other and are also involved in host-commensal relationships, especially regarding the development of the immune system (7, 8, 16). Here, we show that B. thetaiotaomicron produces large amounts of uniformly sized OMVs. Using an optimized methodology for the purification of different membrane fractions, we confirmed that OMVs are highly enriched with lipoproteins, particularly glycosyl hydrolases. Our MS data identified the presence of a lipoprotein exposure sequence (LES) in all OMV-enriched lipoproteins. Employing the α-amylase SusG as a model, we confirmed that the LES is required for surface exposure and showed that it also mediates recruitment into OMVs. To our knowledge, this is the first identification of coupling surface exposure to protein sorting into OMVs.

A few lipoprotein surface transport mechanisms in Gram-negative bacteria have been described (38–41). Shuttling of a group of Neisseria meningitidis lipoproteins to the surface is dependent on proteins Slam1 and Slam2, although the sorting mechanism has not been defined (41, 42). Moreover, the well-studied Bam system for folding of beta-barrel proteins in the outer membrane has been shown to export specific lipoproteins (39). We have not identified orthologs of Slam1 or Slam2 by sequence similarity in any *Bacteroides* genomes. The existence of LES motifs in B. thetaiotaomicron lipoproteins, together with the discovery of the LES in *C. canimorsus*, suggests the existence of a conserved phylum-wide mechanism that flips specific lipoproteins toward the extracellular milieu among *Bacteroidetes*. Defining a functional LES in *Bacteroides* spp. constitutes the first step toward the identification of the machinery that mediates lipoprotein surface exposure in these organisms. Our experiments confirmed that the two Asp residues in positions +3 and +4 respecting the lipid attachment site (+1) are essential components of SusG LES. The proposed B. thetaiotaomicron LES motif CS(D/E)_2_ is similar but not identical to the K-(D/E)_2_ or Q-A-(D/E)_2_ LES proposed for *C*. *canimorsus*. An exhaustive mutagenesis analysis of multiple proteins from several species will be required to exactly define a consensus sequence for the LES motif among *Bacteroidetes*.

**FIG 9:**
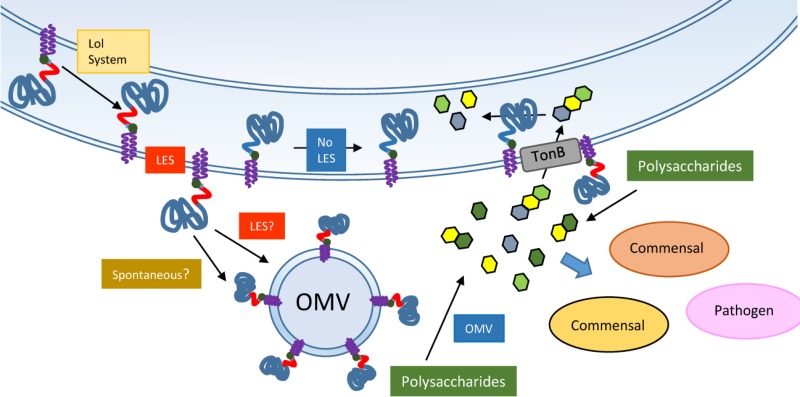
A model for SusG LES-mediated surface exposure and packing into OMV. The SusG lipoprotein is probably transported to the OM by a machinery homologous to the LOL system. The presence of a LES sequence mediates the surface exposure of SusG and its incorporation into OMVs. The LES might have a dual role, where it is required for both surface exposure and OMV packing, or it might be involved only in surface exposure; biophysical properties of lipoproteins would be determinant for packing into OMVs. Surface-exposed SusG in OMVs as well as in the OM hydrolyzes starch molecules into oligosaccharides that can be imported by TonB-dependent receptors by the OMV-producing cell, as well as other commensal and pathogen organisms.

MS results show that OMV-enriched lipoproteins contain a LES motif, suggesting that most if not all OMV-enriched lipoproteins are surface exposed in a LES-dependent manner (Fig. 9). We can speculate with two models to account for this correlation between surface exposure and OMV recruitment of lipoproteins. One possibility is that the LES sequence is somehow recognized by a putative OMV sorting machinery, packaging them preferentially into vesicles. Alternatively, exposed lipoproteins might be recruited into OMVs based on their biophysical properties. Uncoupling surface exposure with OMV packaging is currently not possible, and future work will be required to unravel the molecular basis of the link between these two processes.

In the current models for PUL systems, the hydrolytic enzymes and the oligosaccharide importers are present at the outer membrane (20, 36). However, we determined that functional glycosyl hydrolases are packed mainly into OMVs. We also found that the glycosidases present in OMVs can provide substrates to support the growth of other bacteria, in agreement with the previously proposed function of OMVs as public goods (25, 26). We propose a model in which, in a process involving the LES, the glycosidases are sequentially surface exposed and sorted into OMVs (Fig. 9). The secreted OMVs, armed with an arsenal of hydrolases, can digest diverse dietary polysaccharides and host glycoconjugates, making the mono- and oligosaccharides available to all members of the microbiota. Depending on the diet composition, *Bacteroides* spp. can induce and package different enzyme repertoires, and therefore, members of the microbiota can act as donors or acceptors in their ecological niches. In addition, pathogens such as *Campylobacter*, *Salmonella*, and *Clostridium* can also benefit from the glycosidic activity contained in OMVs (43, 44). From an evolutionary and social standpoint, it is not clear how beneficial OMV-mediated hydrolysis is for the producing organisms. On one side, it is expected that hydrolytic OMVs make the nearby gut environment more mixed in its nutritional composition, rendering the producers more susceptible to cheating by consumers. On the other side, it is tempting to speculate that OMVs might have counteracting benefits to the producers, such as a higher efficiency of hydrolysis than that of OM-anchored enzymes (by increasing the enzymatic surface) and, potentially, an increase in the intestinal surface that could be colonized by its being able to access substrates through a longer stretch of the colonic niche.

We have identified a set of OMV- and OM-enriched proteins that may be employed as markers to investigate the process of OMV biogenesis through the visualization of OMV in the mammalian gut. For example, these markers would allow the differentiation *in vivo* between bona fide OMV and cell lysis-derived material. Furthermore, the identification for the first time of a role for the LES as a signal for protein transport into OMVs constitutes a starting point for the design of novel probiotic interventions in animal and human health. In the future, the LES of SusG or other OMV proteins might be employed to engineer *Bacteroides* strains to secrete OMVs packed with medically relevant proteins in the mammalian gut.

## MATERIALS AND METHODS

### Bacterial strains and growth conditions.

Oligonucleotides, strains, and plasmids are described in Table S3 in the supplemental material. *Bacteroides* strains were grown in an anaerobic chamber (Coy Laboratories) using an atmosphere of 10% H_2_, 5% CO_2_, 85% N_2_. For liquid growth, tryptone-yeast extract-glucose (TYG), TYM (TY medium supplemented with 0.5% maltose instead of 0.5% glucose), or minimal media supplemented with 0.5% glucose or 0.5% potato starch were prepared as previously described (17, 45). Brain heart infusion (BHI) agar with 10% defibrinated horse blood was used as a solid medium. Antibiotics were used as follows: ampicillin at 100 μg/ml, erythromycin at 25 μg/ml, and bromodeoxyuridine at 200 μg/ml.

10.1128/mSphere.00559-18.7 TABLE S3 Oligonucleotides, strains, and plasmids. Download Table S3, XLSX file, 0.0 MB. Copyright © 2018 Valguarnera et al. 2018 Valguarnera et al. This content is distributed under the terms of the Creative Commons Attribution 4.0 International license.

### Construction of plasmids, mutagenesis, and generation of the *susC*-3×Flag strain.

Constructs for overexpression were built on pFD340 (46). Inserts obtained by PCR were digested with restriction enzymes, purified, and ligated onto the digested vector pFD340 as previously reported. For all 6×His-containing constructs, the tag was added to the C terminus of the ORF product by including the 6×His sequence in the reverse primer used for cloning into pFD340 (Table S3). In the cases of BT_0418 and BT_2844, we detected that the putative correct start codons extend 9 and 10 amino acids into the N terminus of the sequence, respectively, and included that information for the cloning of both genes. For cloning of BT_0766-Flag into pFD340, purified PCR inserts were integrated into PCR-amplified pFD340 using an In-Fusion cloning kit (Clontech). Mutagenesis of pFD340/*susG*-6×His was carried out by inverse PCR with *Pfu* Turbo (Agilent) using overlapping oligonucleotides carrying the mismatch required as described elsewhere. Generation of a *susC*-3xFlag strain was carried out using the B. thetaiotaomicron Δ*tdk* strategy as previously described (47). Briefly, 1,000-bp upstream and downstream fragments of a C-terminal *susC*-3xFlag translational fusion were cloned into pExchange-*tdk*. Constructs were conjugated into B. thetaiotaomicron Δ*tdk* cells using previously transformed Escherichia coli S17-1 λ*pir* as a donor, and strain plating and selection were performed as previously described (47).

### OMV preparations.

Outer membrane vesicles were purified by ultracentrifugation of filtered spent media as previously described by our group (5). For MS analysis, OMV preparations were resuspended in 50 mM HEPES, pH 7.4, and *N*-lauroyl sarcosine was added to a 1% final concentration in 1.5 ml polyallomer tubes (Beckman Coulter). Samples were incubated with gentle rocking for 1 h at room temperature (RT) and ultracentrifuged at 100,000 × *g* for 2 h at RT. Supernatants were recovered (OMV-S) and pellets (OMV-P) were resuspended in 50 mM HEPES, pH 7.4. Protein content was quantified using a DC protein assay kit (Bio-Rad). Fractions were lyophilized for MS analysis.

### Membrane preparations.

Total membrane preparations were performed by cell lysis and ultracentrifugation as previously described (5). For separation of the inner membrane (IM) and OM, total membranes were resuspended in 50 mM HEPES, pH 7.4, using a 2-ml glass tissue grinder with a polytetrafluoroethylene (PTFE) pestle (VWR), and *N*-lauroyl sarcosine was added to a 1% final concentration in 1.5-ml polyallomer tubes (Beckman Coulter). Samples were incubated with gentle rocking for 1 h at RT and ultracentrifuged at 100,000 × *g* for 2 h at RT. Supernatants were recovered (IM) and pellets were resuspended in 50 mM HEPES, pH 7.4. Protein content was quantified using a DC protein assay kit (Bio-Rad). Fractions were lyophilized for MS analysis.

### Mass spectrometry analysis. (i) Protein cleanup and in-solution digestion.

Lyophilized protein preparations were solubilized in lysis buffer (4% SDS, 10 mM, 100 mM Tris, pH 8.5) by boiling them for 10 min, and the protein content was assessed by the bicinchoninic acid (BCA) protein assay according to the manufacturer’s instructions. Three hundred micrograms of each preparation and biological replicate was adjusted to a volume of 200 μl and precipitated overnight using 800 μl of ice-cold acetone (1:4, vol/vol). Samples were spun down at 16,000 × *g* for 10 min at 0°C to the resulting protein precipitate, and acetone was removed. Residue acetone was driven off at 70°C for 5 min. Protein precipitants were resuspended in 6 M urea, 2 M thiourea, 40 mM NH_4_HCO_3_ and reduced/alkylated prior to digestion with Lys-C (1/200, wt/wt) and then trypsin (1/50, wt/wt) overnight as previously described (48). Digested samples were acidified to a final concentration of 0.5% formic acid and desalted with using C_18_ stage tips (49, 50).

### Label-free quantification (LFQ)-based quantitative proteomic LC-MS.

Prepared purified peptides were resuspended in buffer A* (2% acetonitrile, 0.01% trifluoroacetic acid) and separated using a two-column chromatography setup composed of a PepMap100 C_18_ 20-mm by 75-μm trap column and a PepMap C_18_ 500-mm by 75-μm analytical column (ThermoFisher Scientific). Samples were concentrated onto the trap column at 5 μl/min for 5 min and infused into an Orbitrap Q-Exactive Plus mass spectrometer (ThermoFisher Scientific) at 300 nl/min via the analytical column using a Dionex Ultimate 3,000 ultrahigh-performance liquid chromatograph (UPLC) (ThermoFisher Scientific). One-hundred-twenty-five-minute gradients were run, altering the buffer composition from 1% buffer B to 28% buffer B over 95 min, from 28% buffer B to 40% buffer B over 10 min, and then from 40% buffer B to 100% buffer B over 2 min; the composition was held at 100% buffer B for 3 min and then dropped to 3% buffer B over 5 min and held at 3% buffer B for another 10 min. The Q-Exactive mass spectrometer was operated in a data-dependent mode, automatically switching between the acquisition of a single Orbitrap MS scan (60,000 resolution) and 15 MS-MS scans (Orbitrap HCD; 35,000 resolution; maximum fill time, 110 ms; and AGC [automatic gain control] at 2 × 10^5^).

### Mass spectrometry data analysis.

Identification and LFQ analysis were accomplished using MaxQuant (v1.5.3.1) (51). Searches were performed against the B. thetaiotaomicron (strain ATCC 29148/VPI-5482) proteome (UniProt proteome identifier UP000001414, downloaded 20 May 2017, 4,782 entries) with carbamidomethylation of cysteine set as a fixed modification and with variable modifications of oxidation of methionine and acetylation of protein N termini. Searches were performed with trypsin cleavage specificity allowing 2 miscleavage events, with a maximum false discovery rate (FDR) of 1.0% set for protein and peptide identifications. To enhance the identification of peptides between samples, the Match Between Runs option was enabled with a precursor match window set to 2 min and an alignment window of 10 min. For label-free quantitation, the MaxLFQ option within MaxQuant (52) was enabled in addition to the requantification module. The resulting protein group output was processed within the Perseus (v1.4.0.6) (53) analysis environment to remove reverse matches and common protein contaminates prior. For LFQ comparisons, missing values were imputed using Perseus. Visualization was done using Perseus and R. Predicted localization and topology analysis for proteins identified by MS was performed using LipoP and TOPCONS (54, 55).

### SDS-PAGE and OMV/OM ratio determination.

Membrane and OMV fractions were analyzed by standard 10 to 12% Tris-glycine SDS-PAGE, as described elsewhere. Briefly, 10 μg of each fraction (transmembrane [TM]/IM/OM/OMV) was loaded onto an SDS-PAGE gel and transferred onto a nitrocellulose membrane, and Western blotting was performed using the LI-COR system. Membranes were blocked using Tris-buffered saline (TBS)-based Odyssey blocking solution (LI-COR). Primary antibodies used in this study were rabbit polyclonal anti-His (ThermoFisher), mouse monoclonal anti-Flag M2 (Sigma), mouse monoclonal anti-E. coli RNApol subunit alpha (Biolegend), and mouse polyclonal anti-SusD/E/F/G (Nicole Koropatkin/Eric Martens, University of Michigan). Secondary antibodies used were IRDye anti-rabbit 780 and IRDye anti-mouse 680 antibodies (LI-COR). Imaging was performed using an Odyssey CLx scanner (LI-COR).

For validation of MS data, duplicate SDS-PAGE gels were stained with Coomassie blue as described elsewhere, and gel images were acquired to determine fraction quality and relative abundance (Fig. S2 and S3). For OMV/OM determination of SusG-6×His experiments, cells were grown in minimal medium with glucose and fractions were prepared as described previously for OMV and membrane preparations in this section. After transference of the preparations to SDS-PAGE gels, nitrocellulose membranes were incubated with Revert total protein stain as described by the manufacturer (LI-COR) and imaged immediately at 680 nm (Fig. S4). After the imaging, Western blotting using SusG antibodies was carried out and membranes were scanned at 780 nm. Total intensity values were calculated for each lane using the Odyssey scanner software (Image Studio; LI-COR). Total intensity values for each fraction, determined by scanning each lane of the Revert stain image, were used to relativize each SusG signal. Relativized OMV and OM SusG fluorescence signals were used to calculate an OMV/OM SusG ratio. Such a ratio was considered 100% for the WT LES SusG construct. Statistical significance between different OMV/OM ratios was determined by an unpaired *t* test for each pair of SusG LES variants.

10.1128/mSphere.00559-18.2 FIG S2 Total protein profiles of membrane and OMV fractions from OMV-enriched protein validations. We performed membrane and OMV fractionation as indicated using cultures grown overnight in TYG medium. Duplicate SDS-PAGE gels loaded with 10 μg of each protein fraction were analyzed for their total protein profiles by Coomassie blue staining. Provided are representative gels used for two OMV-enriched proteins, BT_3960 and BT_1488. Download FIG S2, TIF file, 1.8 MB. Copyright © 2018 Valguarnera et al. 2018 Valguarnera et al. This content is distributed under the terms of the Creative Commons Attribution 4.0 International license.

10.1128/mSphere.00559-18.3 FIG S3 Total protein profiles of membrane and OMV fractions from OM-enriched protein validations. We performed membrane and OMV fractionation as indicated using cultures grown overnight in TYG medium. Duplicate SDS-PAGE gels loaded with 10 μg of each protein fraction were analyzed for their total protein profiles by Coomassie blue staining. Provided are representative gels used for two OM-enriched proteins, BT_0418 and BT_2844. Download FIG S3, TIF file, 1.6 MB. Copyright © 2018 Valguarnera et al. 2018 Valguarnera et al. This content is distributed under the terms of the Creative Commons Attribution 4.0 International license.

10.1128/mSphere.00559-18.4 FIG S4 Total protein stain analysis of fractions for OMV/OM ratio determination. We performed membrane and OMV fractionation as indicated using cultures grown overnight in minimal medium with glucose. SDS-PAGE gels loaded with 10 μg of each protein fraction were transferred onto nitrocellulose membranes and subjected to Revert total protein staining. Membranes were imaged immediately, and Western blot analysis was carried out using anti-SusG antibodies as described in Materials and Methods. The gel shown is representative of all the variants of SusG LES that were assayed for OMV/OM ratio determinations. Download FIG S4, TIF file, 1.0 MB. Copyright © 2018 Valguarnera et al. 2018 Valguarnera et al. This content is distributed under the terms of the Creative Commons Attribution 4.0 International license.

### Proteinase K assays.

Strains from B. thetaiotaomicron were grown in minimal medium with glucose, and cells were washed with phosphate-buffered saline (PBS) and normalized to an optical density at 600 nm (OD_600_) of 9/ml. PBS (540 μl) and 10 μl of a proteinase K (ProK) solution (20 mg/ml) were added to 450 μl of the cell suspension. Tubes were incubated at 37°C, and 200-μl aliquots were removed at different time points and precipitated using trichloroacetic acid (TCA; final concentration, 20% [vol/wt]). Precipitated aliquots were washed twice with acetone, and pellets were resuspended into Laemmli buffer for Western blot analysis. A nontreated control of the cell suspension was incubated for the longest time point of the experiment and TCA precipitated as described above. A similar procedure was followed for OMV ProK treatments; 450 μl of purified OMVs was treated under the same conditions as whole cells.

### Growth curves and OMV complementation.

For growth curves, wild-type or Δ*susG* strains were grown overnight in TYG medium. Cultures were washed with minimal medium (MM) without any carbon source and normalized by their OD_600_ values. Minimal medium with 0.5% starch as the only carbon source was inoculated with WT or Δ*susG* strains to a final OD_600_ of 0.05. OMVs were purified from Δ*susG* strains containing different pFD340/*susG*-6×His derivatives grown in minimal medium with glucose and were added to the Δ*susG* cultures in minimal medium with starch (1 µg/ml final OMV concentration). Aliquots were taken at different times, and the OD_600_ was measured to determine growth. Statistical significance when two growth curves were compared was determined by performing one pair of an unpaired *t* test analysis per each time point.

### Transmission electron microscopy.

For negative staining and analysis by transmission electron microscopy, bacterial suspensions in PBS were allowed to absorb onto freshly glow-discharged Formvar/carbon-coated copper grids for 10 min. Grids were washed in distilled H_2_O and stained with 1% aqueous uranyl acetate (Ted Pella, Inc., Redding, CA) for 1 min. Excess liquid was gently wicked off, and grids were allowed to air dry. Samples were viewed on a JEOL 1200EX transmission electron microscope (JEOL United States, Peabody, MA) equipped with an 8-megapixel digital camera (Advanced Microscopy Techniques, Woburn, MA).

### Data availability.

The mass spectrometry proteomics data have been deposited in the ProteomeXchange Consortium via the PRIDE partner repository with the data set identifier PXD011378 (57).
