# Supplementary figures and images for: Surface Exposure and Packing of Lipoproteins into Outer Membrane Vesicles Are Coupled Processes in Bacteroides
Source: mSphere. 2018 Nov 7;3(6):e00559-18. doi: 10.1128/mSphere.00559-18 (PMC6222051; doi:10.1128/mSphere.00559-18)

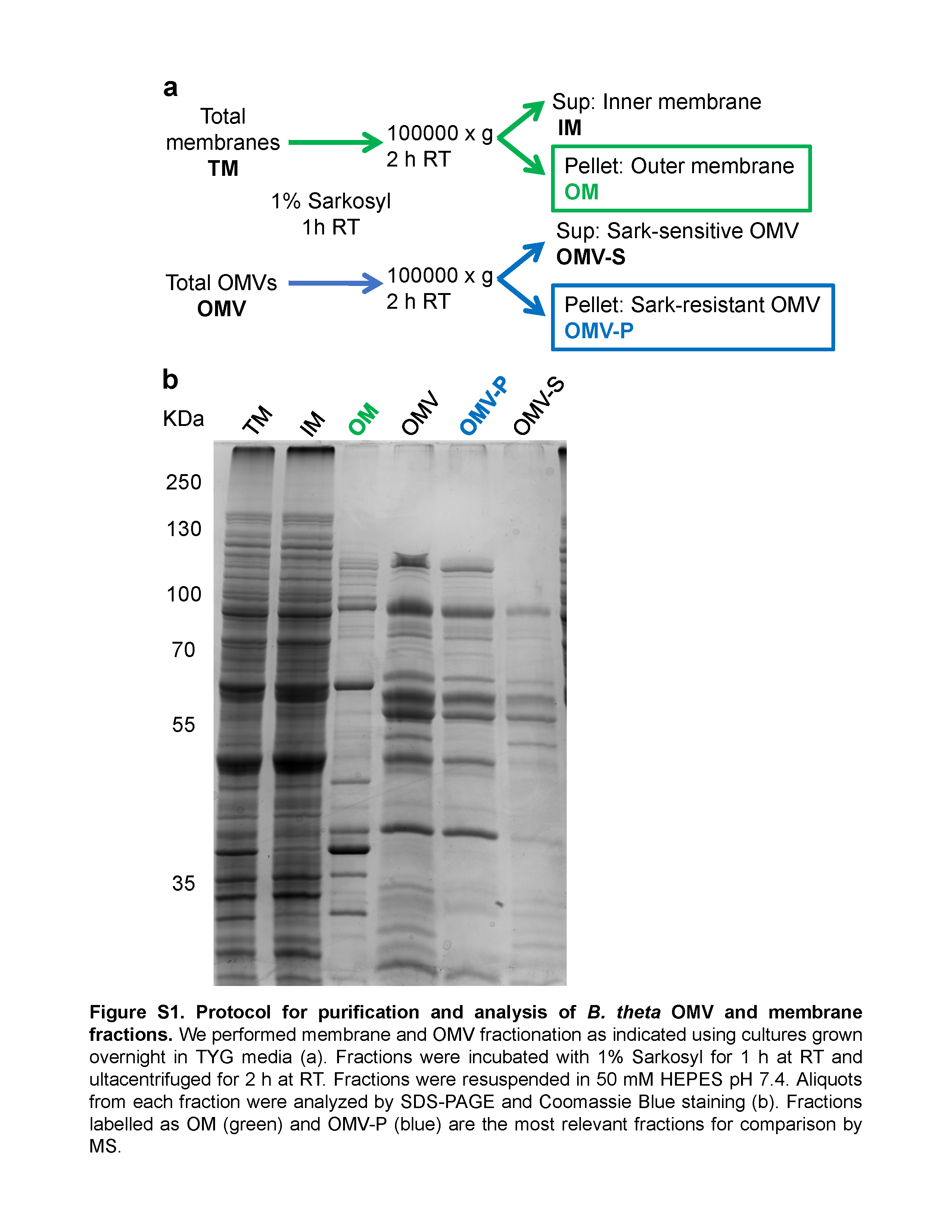

Supplement: FIG S1 [file sph004182696sf1.tif]

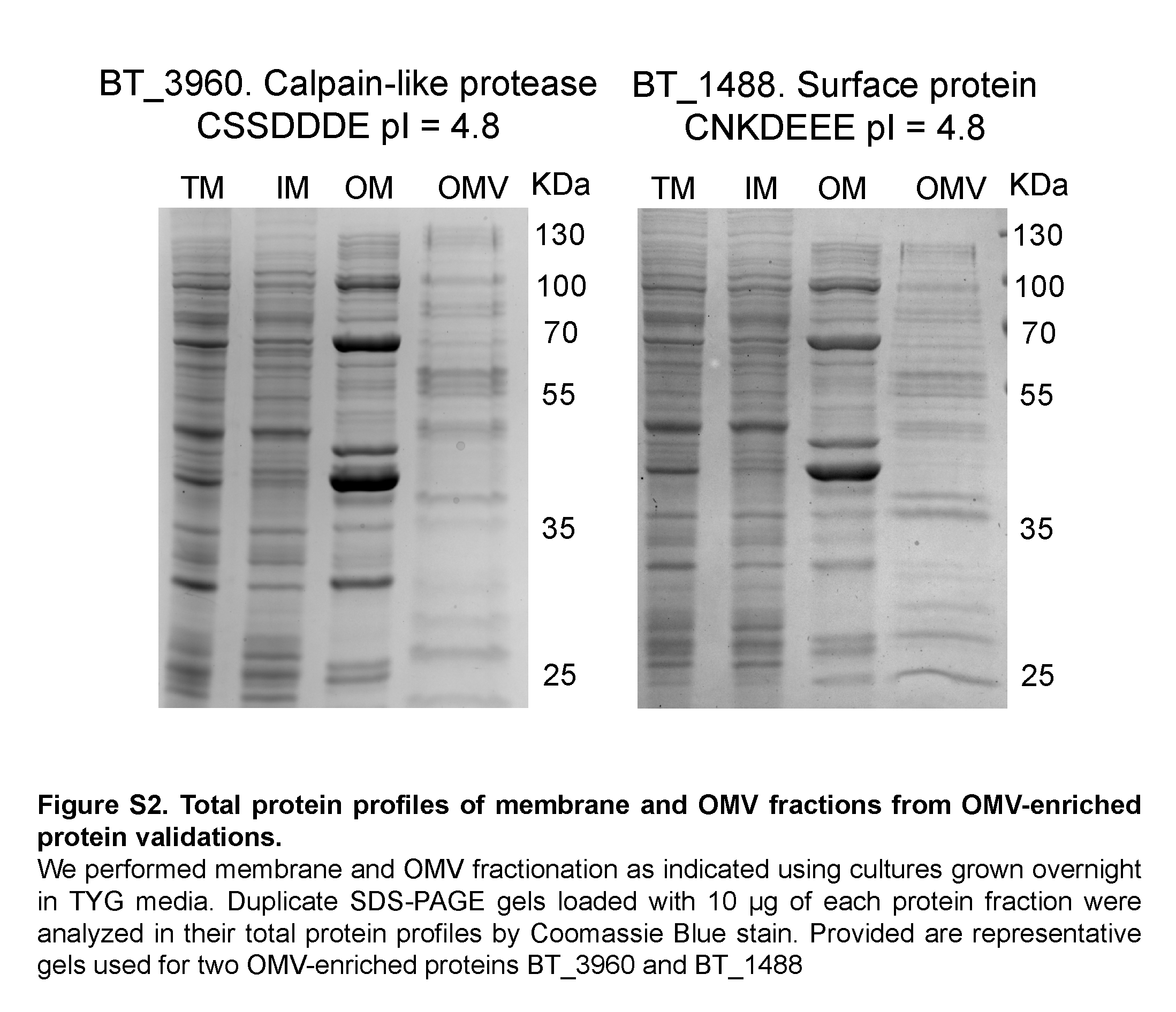

Supplement: FIG S2 [file sph004182696sf2.tif]

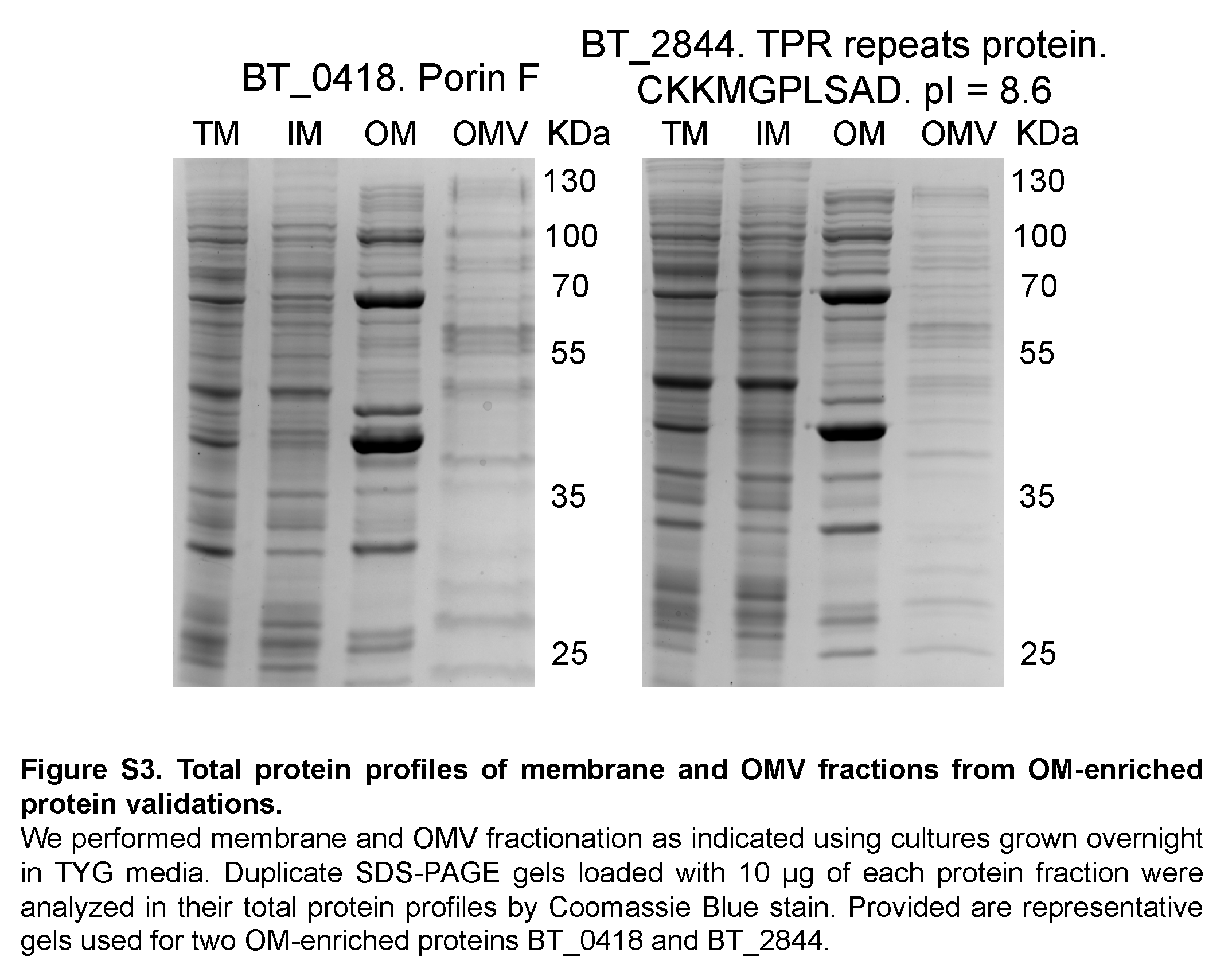

Supplement: FIG S3 [file sph004182696sf3.tif]

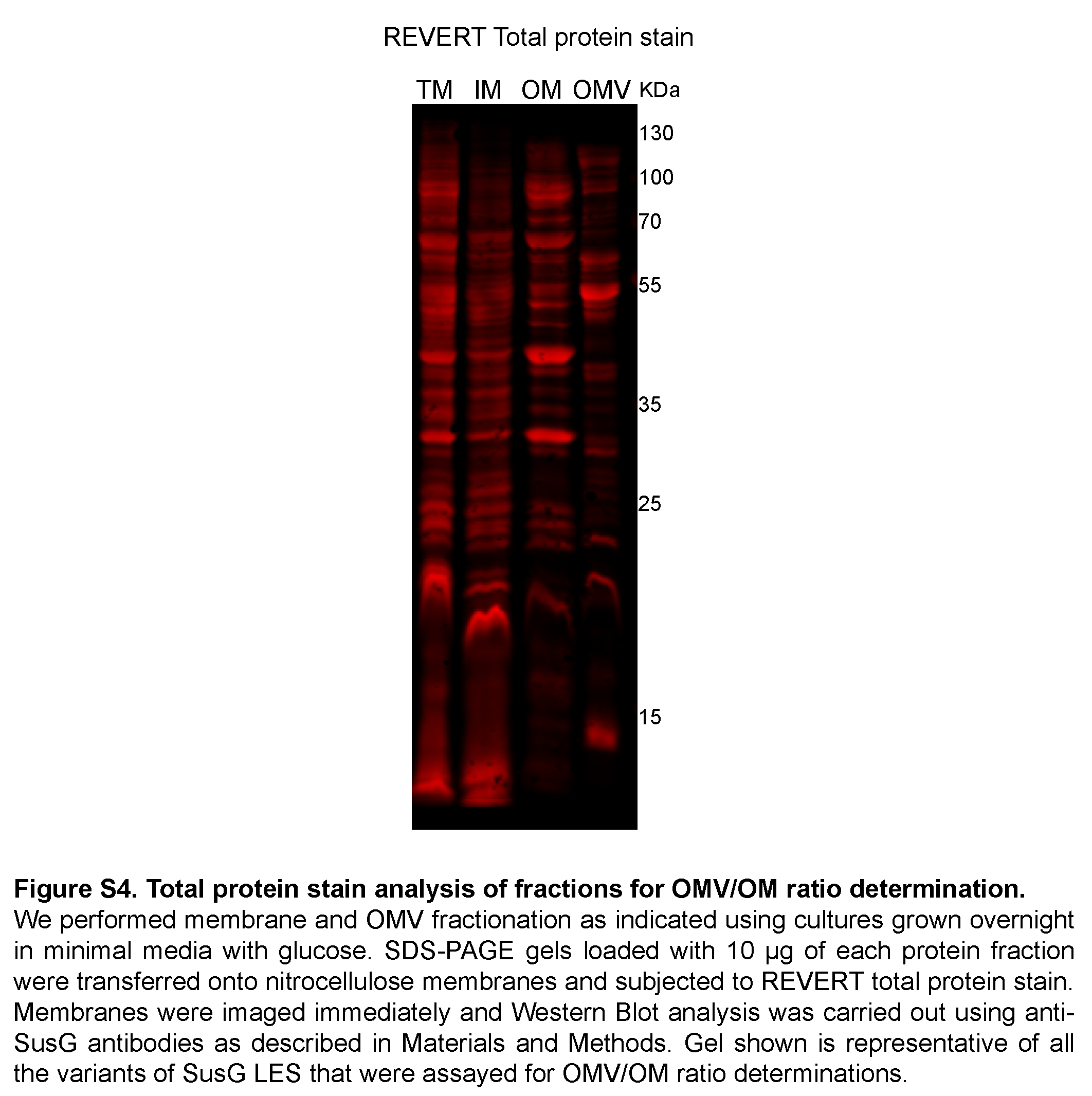

Supplement: FIG S4 [file sph004182696sf4.tif]
